# The structure of volcanic cristobalite in relation to its toxicity; relevance for the variable crystalline silica hazard

**DOI:** 10.1186/1743-8977-9-44

**Published:** 2012-11-19

**Authors:** Claire J Horwell, Benedict J Williamson, Ken Donaldson, Jennifer S Le Blond, David E Damby, Leon Bowen

**Affiliations:** 1 Institute of Hazard, Risk & Resilience, Department of Earth Sciences, Durham University, Science Labs., South Road, Durham, DH1 3LE, UK; 2 Camborne School of Mines, College of Engineering, Mathematics and Physical Sciences, University of Exeter, Cornwall Campus, Penryn, TR10 9EZ, UK; 3 Department of Earth Sciences, Natural History Museum, Cromwell Road, London, SW7 5BD, UK; 4 The Queen's Medical Research Institute, The University of Edinburgh/MRC Centre for Inflammation Research, 47 Little France Crescent, Edinburgh, EH16 4TJ, UK; 5 Brighton and Sussex Medical School, University of Sussex, Brighton, East Sussex, BN1 9PX, UK; 6 Durham GJ Russell Microscopy Facility, Durham University, Durham, DH1 3LE, UK

**Keywords:** Cristobalite, Volcano, Respiratory health, Crystalline silica, Volcanic ash, Soufrière Hills, Quartz, Variable hazards, Regulations

## Abstract

**Background:**

Respirable crystalline silica (RCS) continues to pose a risk to human health worldwide. Its variable toxicity depends on inherent characteristics and external factors which influence surface chemistry. Significant population exposure to RCS occurs during volcanic eruptions, where ashfall may cover hundreds of square km and exposure may last years. Occupational exposure also occurs through mining of volcanic deposits. The primary source of RCS from volcanoes is through collapse and fragmentation of lava domes within which cristobalite is mass produced. After 30 years of research, it is still not clear if volcanic ash is a chronic respiratory health hazard. Toxicological assays have shown that cristobalite-rich ash is less toxic than expected. We investigate the reasons for this by determining the physicochemical/structural characteristics which may modify the pathogenicity of volcanic RCS. Four theories are considered: 1) the reactivity of particle surfaces is reduced due to co-substitutions of Al and Na for Si in the cristobalite structure; 2) particles consist of aggregates of cristobalite and other phases, restricting the surface area of cristobalite available for reactions in the lung; 3) the cristobalite surface is occluded by an annealed rim; 4) dissolution of other volcanic particles affects the surfaces of RCS in the lung.

**Methods:**

The composition of volcanic cristobalite crystals was quantified by electron microprobe and differences in composition assessed by Welch’s two sample *t*-test. Sections of dome-rock and ash particles were imaged by scanning and transmission electron microscopy, and elemental compositions of rims determined by energy dispersive X-ray spectroscopy.

**Results:**

Volcanic cristobalite contains up to 4 wt. % combined Al_2_O_3_ and Na_2_O. Most cristobalite-bearing ash particles contain adhered materials such as feldspar and glass. No annealed rims were observed.

**Conclusions:**

The composition of volcanic cristobalite particles gives insight into previously-unconsidered inherent characteristics of silica mineralogy which may affect toxicity. The structural features identified may also influence the hazard of other environmentally and occupationally produced silica dusts. Current exposure regulations do not take into account the characteristics that might render the silica surface less harmful. Further research would facilitate refinement of the existing simple, mass-based silica standard by taking into account composition, allowing higher standards to be set in industries where the silica surface is modified.

## Background

Exposure to respirable crystalline silica (RCS) has been known for centuries to be detrimental to health, and is linked to respiratory diseases such as silicosis, lung cancer and tuberculosis. However, when classifying crystalline silica as a Group 1 carcinogen, the IARC Working Group recognised that silica dusts are variably hazardous and that their carcinogenicity may depend on inherent characteristics of the silica, and/or external factors which affect the particle surface and biological activity [1]. The UK Health & Safety Executive assessed the potential of RCS to cause silicosis and concluded that all forms of RCS dusts of occupational relevance have this potential but that, again, the fibrogenicity will be influenced by the physicochemical characteristics of the dust [2].

Crystalline silica occurs as five main polymorphs with quartz, cristobalite and tridymite being more reactive and cytotoxic than coesite and stishovite [2]. Quartz is the most common mineral in the continental crust and is the most commonly-encountered polymorph. Occupational exposure to the other polymorphs is more unusual but cristobalite exposure occurs in the ceramics industry through conversion of quartz in industrial furnaces [3], and through crystallization from amorphous diatomaceous earth during calcination [4]. *In vitro* experiments have suggested that cristobalite and quartz are comparably cytotoxic, inflammogenic and fibrogenic [5-8].

In this paper, we investigate the structural side of the ‘structure-toxicity relationship’ for RCS generated during volcanic eruptions. Volcanoes are a major source of natural silica and 9% of the world’s population lives within 100 km of a historically active volcano [9]. The discovery of substantial quantities of RCS in volcanic ash from both the 1980 eruption of Mt St Helens, USA and the eruption of Soufrière Hills volcano (SHV), Montserrat, West Indies (1995 onwards), resulted in widespread concern regarding exposure to volcanic ash over prolonged durations [10,11]. As volcanoes can mass-produce RCS, in close association with other minerals during eruptions, this presents a unique opportunity to characterise mineralogical factors which could influence respiratory toxicity.

### Formation of cristobalite in volcanic environments

**Figure 1:**
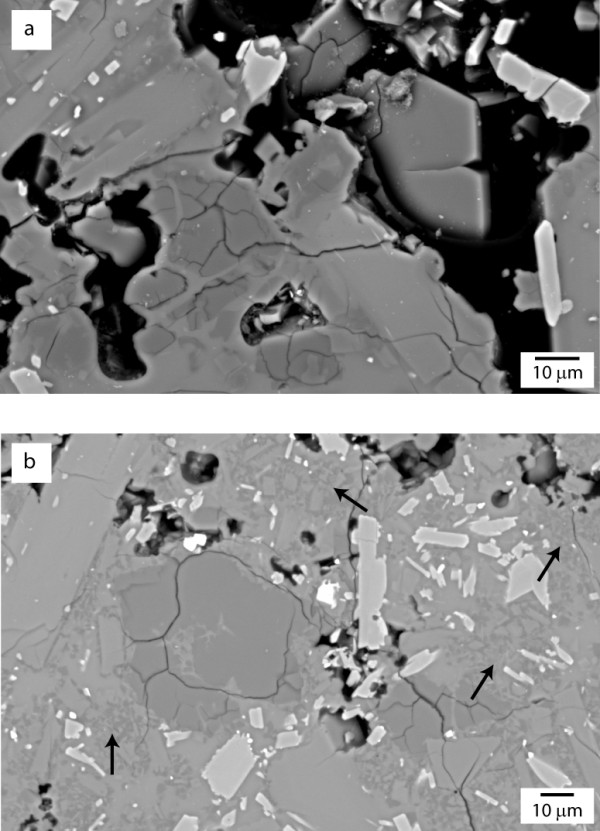
**Backscattered electron (BSE) images showing cristobalite textures in thin section. a**) Cristobalite crystal showing typical ‘fish-scale’ cracking in MVO1236. The boundary between the fish-scale cristobalite and surrounding groundmass is unclear. One platy crystal is protruding from a pore to the right, centre of the image; **b**) ‘Feathery’ groundmass texture (indicated by arrows) in MVO617, composed of cristobalite, glass and plagioclase feldspar (Horwell et al. [12]) with associated fish-scale cristobalite which, in this case, is probably a product of extensive devitrification and also has a diffuse boundary.

The mass formation of crystalline silica in volcanic environments usually follows lava dome eruptions where viscous, silicic magma is extruded from the volcano at ~800°C, forming a dome of rock in the crater. Magmatic vapours circulate through the dome depositing crystalline silica, as cristobalite, in cracks and pore spaces [10,12]. Horwell et al. [12] observed two forms of vapour-phase cristobalite in the pore spaces of SHV dome rock: prismatic crystals and platy, hexagonal crystals. Both display a distinctive ‘fish-scale’ cracked texture when observed in thin section (Figure 1). Volcanic glass in the lava may also devitrify, again forming cristobalite, or quartz if devitrification is influenced by hydrothermal fluids [10,12]. Cristobalite may also form in the volcanic edifice by deposition and alteration from circulating hydrothermal fluids. Lava domes are inherently unstable and frequently collapse, fragmenting the rock and forming pyroclastic density currents (PDCs, also known as pyroclastic flows), which move rapidly down the volcanic flanks. A ‘co-PDC’ ash plume lofts from the PDC, which is enriched in fine particulate and, in particular, RCS [13] and may travel hundreds of kilometres, depositing ash of which ~20-40% is composed of the ‘thoracic’ fraction (< 10 μm) and ~10-20% is ‘respirable’ (< 4 μm) [14]. At SHV, cristobalite comprises up to 23 wt. % of the bulk ash [15]. Quartz may be present in ash from intermediate to felsic (high SiO_2_) volcanoes as crystals which grow in the ascending magma (phenocrysts) on its journey from the magma chamber to the surface. A summary table of the different habits of crystalline silica found at dome-forming volcanoes is found in [12]. 

Fragmentation of the lava produces freshly-fractured mineral surfaces, a factor which is known to increase the surface reactivity and pathogenicity of quartz [16-20] but the effects of which are less well characterised for cristobalite. Horwell et al. [21] showed that the surface reactivity of SHV volcanic ash is substantially reduced in aged, weathered samples, in comparison to fresh ash, and is enhanced in ground samples.

Dome-forming eruptions are often long-lived (e.g. SHV has been erupting since 1995) so it is imperative that the respiratory hazard of volcanic cristobalite is characterised. Although cristobalite has comparable toxicity to quartz, far fewer epidemiological and clinical studies have been carried out on industrial cristobalite exposure to support the laboratory conclusions, mainly because of the limited workplace exposures specific to cristobalite (e.g., pottery and refractory brick workers are usually co-exposed to quartz and cristobalite [1]). The diatomaceous earth industry, however, produces cristobalite from amorphous silica during calcination of diatomite, rather than a conversion from quartz to cristobalite as is the case with ceramics. We have found 24 epidemiological and clinical studies on diatomaceous earth workers in the literature; most have found a dose-response association between cristobalite exposure and lung cancer mortality, non-malignant respiratory diseases (NMRD) mortality and silicosis e.g. [22-25].

At both SHV and Mt St Helens, *in vivo* (inhalation and instillation) and *in vitro* toxicological studies were carried out to determine the likely outcomes of inhaling cristobalite-laden ash reviewed by [26,27]. Differing study designs made comparisons challenging; however, in general, the ash was not found to cause the rapid *in vivo* or *in vitro* response expected of a crystalline silica-rich dust e.g. [28,29]. Of the 19 studies on Mt St Helens ash (9 *in vitro*; 6 instillation and 4 inhalation studies), the exceptions were inhalation studies where very high doses of ash (50-100 mg m^-3^) were administered over long durations (up to 24 months), causing fibrosis and lesions [30,31]. For SHV, it was concluded that the ash may pose a similar health risk to mixed coal mine dust [28]. This conclusion was based on *in vivo* studies carried out over relatively short time periods< 49 weeks e.g. [32], and one must be cautious in interpretation of these data as there is a mean latency period for chronic silicosis of around three decades, depending on exposure [33]. A risk assessment of the long-term, cumulative exposure to cristobalite at SHV concluded that, assuming continuing volcanic activity, the risk of silicosis for gardeners (highest occupational exposure) after 20 years of continuous exposure is 2 – 4% [34], based partly on the toxicology data. Overall, evidence suggests that the ash represents a relatively low hazard. Given the quantities of RCS in the ash, we sought to determine the structural factors that could be masking the toxicity of volcanic cristobalite.

The variability of the RCS hazard in workplaces is still a key issue, especially as regulations take a ‘one size fits all’ approach to crystalline silica [35]. Studies on the RCS hazard, such as the current structural work on volcanic ash, will add to the growing weight of evidence that inherent characteristics and external factors can be measured, which may provide an opportunity to refine RCS exposure limits to take such factors into account.

### Potential factors affecting toxicity

**Figure 2:**
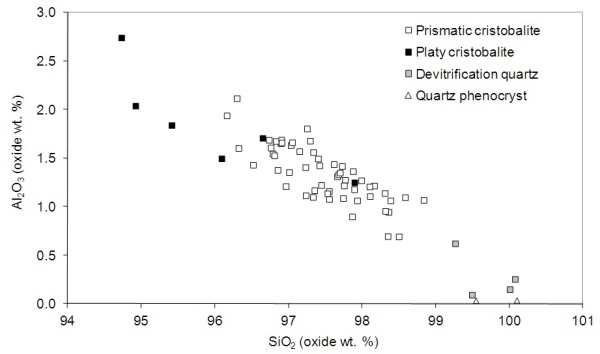
**Al**_**2**_**O**_**3**_**vs. SiO**_**2**_**(wt. %) for prismatic and platy cristobalite, and devitrified and magmatic quartz.** Data derived from electron microprobe analyses of individual crystals for all dome-rock samples. No data for devitrification cristobalite as ‘feathery’ crystals were too small for the resolution of the microprobe.

The pro-inflammatory effects of quartz may be modified by addition of a range of substances that prevent the surface from producing the classic quartz inflammatory response [36,37]. In particular, treatment with Al salts, such as Al lactate, has been shown to lower quartz toxicity [38-40]. A feature of the SHV cristobalite (SiO_2_) is the presence of low levels of aluminium (Al) and sodium (Na) as indicated from SEM energy dispersive X-ray spectroscopy (SEM-EDS) of individual cristobalite ash particles (see minor peaks for Al and Na in addition to the Si and O peaks in the crystalline silica EDS spectrum in Horwell et al. [41] Figure 2). Therefore, the presence of Al and Na in close association with RCS in volcanic ash could be important in passivating cristobalite. We propose four theories for the involvement of these elements in modulating the cristobalite hazard in SHV ash:

1. **Crystal composition.** The reactivity of particle surfaces could be reduced due to co-substitutions of Al and Na for Si in the cristobalite structure as the crystals form. Cristobalite has an open lattice structure which readily permits substitution of Si^4+^ by Al^3+^ with charge balancing by other cations such as Na^+^ or K^+^[42].

2. **Heterogeneous surface.** During dome collapse, fragmetation of dome lava is unlikely to result in the production of fresh cristobalite crystal surfaces. Cristobalite ash particles may have fragments of other volcanic phases (plagioclase, glass, salts and other vapour-phase precipitates) inter-grown or adhered to their surfaces which will decrease the surface area of cristobalite available for reactions in the lung. Both feldspar and glass are Al-rich. The grain boundary between large (> 10 μm) ‘fish-scale’ cristobalite patches and vitreous groundmass is often diffuse, with cristobalite appearing to penetrate and merge with the groundmass [12]. In addition, sub-micron-scale ‘feathery’ cristobalite crystallites, along with plagioclase feldspar, form as devitrification patches within groundmass glass [12].

3. **Annealed surface.** The surface of cristobalite particles could be occluded by an annealed glass layer (known as a Beilby layer [43]). Alternatively (or, in addition), during formation, if crystallization begins above the solidus when pores are still plastic (e.g. [44]) or during fragmentation, cristobalite particles may become coated in a nano-scale layer of amorphous volcanic glass.

4. **Silicate dissolution.** The mixture of cristobalite and other silicate and glass particles, in a heterogeneous dust, may affect the hazard. Proximity of cristobalite to soluble phases such as glass, following lung deposition and phagocytosis, could affect the RCS surface.

Theories 1-3 address ‘inherent characteristics’ of the RCS particles whereas theory 4 addresses ‘external factors’ which may influence the hazard in the lung. In this study we use mineralogical techniques to address theories 1 to 3. To address theories 1 and 2 we investigated 9 samples of SHV dome rock, mainly collected from the Montserrat Volcano Observatory (MVO) archives (see Table 1). Samples are described in detail in Horwell et al. [12]. To address theories 2 and 3 we analysed SHV dome-collapse ash samples, also detailed in Table 1. 

**Table 1:** **Sample summary with information on crystalline silica features observed by Horwell et al.**[12]

**Sample No.**	**Date of collapse/eruption**	**Date of collection**	**Description**	**Eruption Information**	**Crystalline silica type**			
					**Prismatic**	**Platy**	**Devit. cristob.**	**Devit. quartz.**
**Dome rock:**								
MVO819	174 k.a. BP	15/2/98	Dome lava; very dense, grey/brown	Ancient sample from old complex	✓ but rare	✓	✓	✓
MVO945	400 a BP	?	Dome lava; Dense, red/brown	Ancient simple from Castle Peak	✓	×	×	✓
MVO287	21/9/97	21/9/97	Juvenile block; vesicular; light grey	From dome collapse deposit	✓	×	✓	×
MVO288	21/9/97	21/9/97	Juvenile block; frothy, light grey	From dome collapse deposit	✓ but rare	×	×	×
MVO617	21/9/97	16/5/98	Dome lava; Dense; Green/dark grey	From dome collapse deposit	✓	×	✓	×
MVO332	26/12/97	4/1/98	Dome lava; Vesicular; pale grey	From 'Boxing Day' collapse deposit	✓	×	×	×
MVO1236	12/7/03	1/8/03 – 15/11/03?	Dome lava; Dense; pale grey	From dome collapse deposit (full collapse)	✓	✓	✓	✓
MVO1406	20/5/06	?	Dome lava; dense; pale grey	From dome collapse deposit	✓	×	✓	×
MontR1*	20/5/06	22/6/06	Dome lava, dense, altered, red	From dome collapse deposit	✓	✓	×	✓
**Ash:**								
MRA5/6/99	05/6/99	05/6/99	Co-PDC ash	Respirable (< 4 μm) fraction				
MBA12/7/03	12/7/03	12/7/03	Co-PDC ash	Bulk ash from which cristobalite separated				

‘Prismatic’ = euhedral cristobalite; ‘Platy’ = platelets of cristobalite; ‘Devit. cristob.’ = cristobalite during glass devitrification; ‘Devit. quartz’ = quartz formed (probably through phase transition from cristobalite) when the glass is totally devitrified.

*Collected by BW from deposit rather than archive.

MVO sample numbers assigned by the Montserrat Volcano Observatory.

## Results

The extensive collection of well characterised dome rock and ash from the long-lived SHV eruption [12], held at MVO, provides the best archive of material to examine the theories for variable toxicity offered above.

### Elemental composition of cristobalite

From electron microprobe analyses of cristobalite crystals in dome rock, we found that both prismatic and platy cristobalite forms were not pure SiO_2_, containing up to ~ 3 wt. % Al_2_O_3_ (Figure 2 and Table 2). The prismatic cristobalite contained 0.7 - 2.1 wt. % Al_2_O_3_ and the platy cristobalite from 1.3 to 2.7 wt. % Al_2_O_3_. Comparison of these datasets by Welch’s *t*-test yielded a *p*-value of 0.0292, indicating that the null hypothesis – that there is no difference in the data – can be rejected at the 5% level, hence the amount of Al_2_O_3_ in the platy and prismatic cristobalite forms is significantly different. The devitrification quartz (in samples MontR1, MVO1236 and MVO945), had Al_2_O_3_ values of 0.1 to 0.6 wt. %, and two quartz phenocrysts contained minimal Al_2_O_3_.

**Table 2:** Representative electron probe results for cristobalite and quartz phases

	**Prismatic cristobalite**			**Platy cristobalite**			**Devitrification quartz**			**Quartz phenocryst**
	**MVO617**	**MontR1**	**MVO1406**	**MVO819**	**MVO819**	**MVO1236**	**MontR1**	**MontR1**	**MVO945**	**MVO287**
Na_2_O	1.14	0.30	0.41	0.98	0.70	0.96	0.14	n.d.	n.d.	n.d.
Al_2_O_3_	1.94	1.42	0.96	2.74	1.50	1.71	0.62	0.09	0.25	n.d.
SiO_2_	96.16	97.42	98.32	94.73	96.10	96.65	99.27	99.49	100.08	100.10
MgO	n.d.	0.02	n.d.	0.04	0.12	n.d.	0.02	n.d.	n.d.	n.d.
K_2_O	0.01	0.03	0.23	0.60	0.26	n.d.	0.03	0.13	n.d.	0.01
CaO	0.08	0.29	0.02	0.12	0.08	0.06	0.47	0.22	0.05	n.d.
TiO_2_	0.23	0.09	0.17	0.17	0.17	0.15	0.11	0.08	0.08	0.03
MnO	n.d.	n.d.	n.d.	n.d.	n.d.	n.d.	n.d.	n.d.	n.d.	n.d.
FeO	0.20	0.20	0.09	0.34	0.37	0.19	0.16	0.29	0.15	n.d.
Total	99.79	99.77	100.19	99.75	99.27	99.75	100.81	100.12	100.64	100.21

Data are in oxide wt. %. n.d. = not detected.

Sodium was also detectable in cristobalite, at levels up to 1.1 wt. % Na_2_O. The prismatic crystals contained 0 – 1.1 wt. % Na_2_O compared with 0.7 – 1.1 wt. % Na_2_O in the platy crystals. These data are also significantly different at the 5% level (p=0.0301). The platy cristobalite, in general, also contained slightly elevated levels of other major element oxides (e.g. K_2_O, Fe_2_O_3_, CaO and TiO_2_).

### Cristobalite and associated phases

Observations of sectioned dome rock by Scanning Electron Microscopy (SEM) confirmed that cristobalite is intimately inter-grown with surrounding mineral phases and glass (Figure 1a). Additionally, devitrification crystallites of cristobalite are sub-micron sized, and are closely associated with glass and feldspar (composition confirmed by Horwell et al. [12]) (Figure 1b). It was also observed that, whilst whole cristobalite crystals in the dome rock are usually 20-50 μm diameter (i.e. within the ‘inhalable’ sub-100 μm fraction, but larger than the ‘thoracic’ (sub-10 μm) or ‘respirable’ (sub-4 μm) fractions), ‘fish-scale’ cracking divides the cristobalite into roughly sub-10 μm segments (as can be seen in Figure 1a and b).

**Figure 3:**
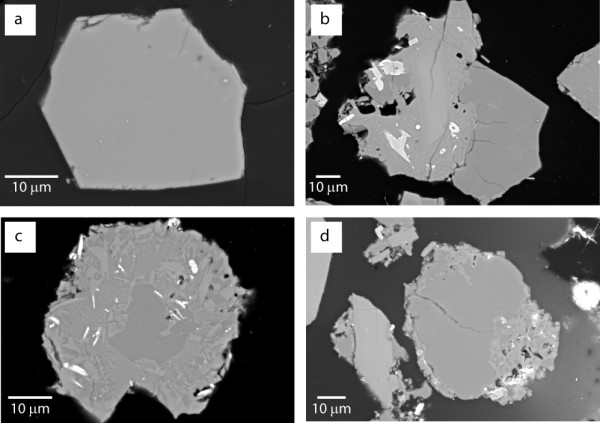
**BSE images of resin mounted and polished cristobalite particles separated from volcanic ash sample MBA12/7/03.a**) A particle composed solely of cristobalite; **b**) A particle composed of a cristobalite crystal still attached to the groundmass patch from which it nucleated; **c**) A particle of ‘feathery’ devitrified groundmass with a totally-devitrified cristobalite patch in the centre; **d**) A particle of cristobalite with a ‘rim’ of glassy groundmass.

Observation of sectioned and polished cristobalite ash particles by SEM showed that: a) some particles are solely composed of cristobalite (Figure 3a); b) some particles are a mixture of cristobalite plus feldspar, glass or other volcanic minerals, either from break up of vapour-phase crystals and associated groundmass (Figure 3b) or devitrification patches (Figure 3c); c) some particles have a cristobalite core but also have patches of other minerals or glass adhered to their surface (Figure 3d). All ‘rims’ appeared to be patches of groundmass which had remained attached to the cristobalite during fragmentation. No cristobalite crystals appeared to be coated with volcanic glass (i.e. melt adhering to crystal surfaces within plastic vesicles prior to solidus).

**Figure 4:**
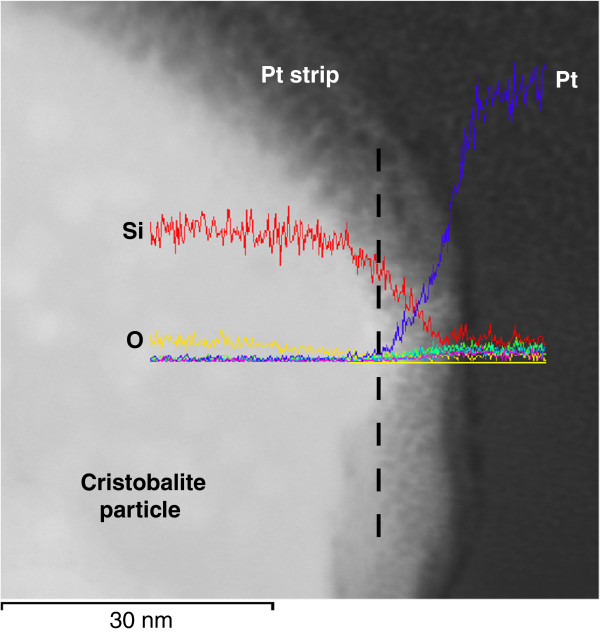
**FEG-TEM image of thinned edge of a cristobalite crystal from volcanic ash sample MRA5/6/99.** The platinum (Pt) strip shows as dark grey on the right hand side of the image with the mottled area being attributed to crystal thinning or background scattering from the Pt strip. The dashed black line represents the approximate boundary between the crystal and the strip. Superimposed on the image are the EDS results from a 50 nm transect from rim towards the core of the crystal. Pt = dark blue; Si = red; Al = green; K = pink; Fe = turquoise; Cl = light blue; O = yellow.

Analysis, by both TEM-EDS and SEM-EDS, did not reveal obvious changes in cristobalite composition within nano- or micro-transects from crystal rim towards crystal core (Figure 4) with no observable higher concentration (or deficit) of Al and other elements at the grain margin. For the TEM-EDS transect, the observed decrease in SiO_2_ towards the rim is attributed to reduced crystal depth towards the edge of the crystal. The minor increase in Al, Fe, Cl and K at the rim correspond exactly to the increase in Pt and are probably associated with an increase in background noise due to the thickness of the Pt strip. We were unable to observe changes in crystallinity by TEM (i.e. a nano-scale amorphous coating resulting either from annealing of the crystal surface or an artefact of the FIB thinning process) towards the samples’ rims. The quartz Beilby layer has been determined to be between 0.03 and 0.8 μm [45,46] thick. 

## Discussion

### Factors affecting cristobalite toxicity in Soufrière Hills ash

Electron microprobe analyses of *in situ* vapour-phase cristobalite crystals in dome rock confirmed that the Soufrière Hills cristobalite is not pure SiO_2_ (theory 1). The fact that Al has been shown to be the dominant substituted cation in cristobalite is critical for understanding its toxic potential.

The toxicological effects of cristobalite surface modification have not been previously studied, but it is known that the structure of cristobalite allows substitutions of Al for Si [42]. The mechanism by which Al interacts with quartz is not well understood though (it does not have the open lattice structure of cristobalite or tridymite); however, in acidic conditions, Al is thought to insert into the quartz crystal lattice [37] at discrete points where defects enable this bonding to occur [47]. Duffin et al. [39] treated laboratory standard DQ12 quartz surfaces with Al lactate which had the effect of inhibiting the production of cytokines from macrophages and epithelial cells. The release of reactive oxygen species, capable of causing mutations in the cells of the lung, a likely mechanism for the development of cancer [48], would also be affected if the silica surface were affected by Al. Analogously, the presence of Al in the cristobalite structure and, therefore, at discrete points at its surface, could have similar effects. To test the toxicity side of this structure-toxicity relationship, we are now synthesising cristobalite in the laboratory with controlled levels of Al and Na. The resulting powders will be exposed to alveolar macrophages and cytotoxic, metabolic and pro-inflammatory effects measured to test our theory that structural substitutions in cristobalite affect toxic response.

These structure substitutions may also control crystal morphology. The platy cristobalite contains significantly more Al and Na than prismatic crystals and, hence, at this level of substitution in the crystal structure, prismatic crystal formation may be inhibited. This leads to the possibility that differing cristobalite morphologies may also be variably pathogenic, depending on their Al content (i.e. platy crystals would be less toxic). However, Horwell et al. [12] did not observe solely platy crystals in these dome rock samples – they were always seen in association with prismatic and devitrification cristobalite (Table 1) - so it is unlikely that exposure would ever be restricted to the less-toxic variety.

A second mechanism for generation of cristobalite particles with heterogeneous surfaces is through dome-collapse fragmentation processes, generating particles which are mixed phases of cristobalite and other minerals (theory 2). Our results show this to be the case, as particles (in cross-section) are rarely observed to be solely comprised of cristobalite. These mixed-phase particles likely derive from: 1) vapour-phase crystals which were attached to (and will have nucleated from) groundmass or phenocrysts adjacent to the pore spaces, so we may expect that at least one surface of the vapour-phase cristobalite may have a 'rim' composed of other materials; or 2) break up of devitrified patches of glass where we would expect to see particles composed of feathery crystallites of cristobalite, plagioclase and a glassy matrix. These associated phases give an additional explanation as to why Horwell et al. [41] found that crystalline silica ash particles contain minor amounts of other elements (by SEM-EDS). It is also likely that many whole particles identified as plagioclase or glass by SEM-EDS e.g. by [41] would contain a crystalline silica component.

We did not observe evidence for any cristobalite surfaces being occluded by a thin coating of volcanic glass, clay, or an annealed amorphous layer (theory 3). In studies assessing the toxicity of coal dust, low-rank coals have been observed to contain crystalline silica coated with clays [49]. The clays, and associated soluble extracts, in the coal mine dust are thought to inhibit the cellular reactivity of quartz [50]. Fresh volcanic ash does not contain clay; however, old ash deposits, which may be quarried for volcanic aggregate, may well contain clays derived from chemical weathering of feldspar. Therefore, studies assessing the toxicity of crystalline silica-rich volcanic-derived quarry dust should assess whether silica occlusion is occurring.

When considering health, we can now say that, for the Soufrière Hills ash, there are two inherent factors which may act to reduce the toxicity of cristobalite: 1) the cristobalite is not pure SiO_2_ (theory 1); 2) heterogeneous RCS surfaces will be produced by fragmentation of devitrified areas and will consist of vapour-phase cristobalite and adhered fragments of groundmass (theory 2). All of these processes involve the introduction of Al to the RCS surface.

This evidence of surface modification and heterogeneity provides an explanation as to why the predicted toxicity is not observed in reality e.g. [28,29] and also why SHV ash does not generate silica surface radicals (e.g., Si**·** and Si–O**·**) [21]. However, there is also evidence here for the formation of reactive RCS volcanic particulate; the cracked, ‘fish-scale’ cristobalite provides weakened planes for the generation of inhalable sub-particles (< 15 μm diameter) of cristobalite on fragmentation and it appears that these surfaces remain exposed (i.e. no glass coating). The exposed crack intersections and twin planes at the surface of a crystal can be highly reactive [18] and may host transition metal ions, giving a possible source area for hydroxyl radical activity (HO**·**) [21]. In addition, in areas of extensive devitrification, cristobalite can recrystallize to quartz, which may form up to 5 wt. % of dome rock in altered samples [12]. The toxicity of quartz particles may be different to cristobalite and, therefore, a variably-altered volcanic dome may produce ash with a range of toxicities.

Considering external factors, the heterogeneity of volcanic ash, with the inevitable close association of cristobalite and Al-rich mineral particles (such as feldspar) in the lung could also contribute to impairment of RCS toxicity (theory 4). Diffusion of elements from dissolving/leaching particles to the RCS surface is a possibility on the lung surface but this is likely to be greatly increased within the acidic lysosomal environment following phagocytosis by macrophages, where several particles may be stored in close proximity. In industry, quartz is also extracted with other minerals (e.g. clays), for example, during quarrying and industrial processing of sedimentary rock. For coal-mine dust, it is believed that, where quartz levels are below 10 wt. %, the total mass dust exposure is the best correlate with coal workers’ pneumoconiosis (CWP) [51,52]. However, above 10 wt. %, silicosis and rapid progression of CWP may occur [53]. The lack of silica reactivity at low concentrations cannot only be due to occlusion of RCS surfaces by clays, as discussed above. When DQ12 quartz is pre-incubated with an extract of coal-mine dust, haemolysis drops from 90% in non-incubated DQ12 quartz to virtually zero in incubated samples, demonstrating that there is an external control, such as diffusion of ions to the quartz surface [50].

Determining if volcanic cristobalite toxicity is affected by external factors such as accompanying minerals is challenging as volcanic cristobalite cannot be isolated from the other minerals without affecting the surfaces of the particles (for example through heavy liquid separation or boiling the ash in phosphoric acid). Additionally, several minerals in volcanic ash are iron rich (e.g. amphiboles, pyroxenes and oxides) and Horwell et al. [21,54] have shown that deleterious, iron-catalysed hydroxyl radicals can be generated by these minerals in Soufrière Hills ash, providing a possible separate mechanism for toxicity.

### The variable hazard of RCS

Since IARC classified crystalline silica as a carcinogen, with the proviso that hazard was likely to vary depending on the characteristics of the dust [1], many authors have attempted to quantify the variability of the silica hazard. Studies have shown how cellular responses related to inflammation and fibrosis vary amongst different polymorphs and dust sources, and even amongst samples from the same source e.g. [48,55]. Donaldson and Borm [56] have clarified that the surface reactivity of quartz is key, and that the ability of particles to induce oxidative stress could be modified by substances which affect its surface, including those derived from accompanying minerals. Therefore, toxicity could vary dramatically depending on the origin of the silica. Other authors have suggested that transient piezoelectric charges on freshly-fractured surfaces or the hydrophilicity of the surface may play a role in reactivity [57,58].

In this study, we have investigated the mineralogical features which may modulate RCS toxicity. In the case of volcanic ash, we have shown evidence that the toxicity of RCS is likely affected by ‘inherent characteristics’ of the silica and hypothesized that ‘external factors’ may also act to hinder the reactivity of RCS. However, Fubini [37], postulated that the inherent characteristics and external factors may be active at different stages in disease development, or be involved in different disease pathways. There is also evidence that different disease mechanisms (e.g. inflammation and DNA damage) involve different properties of the silica particle [59,60].

In occupational settings, RCS surfaces may be exposed and activated by grinding or milling [20,61,62], modified by the environment in which they are ground [18] and altered by heating (e.g., for fly ash and biogenic silicas) [63,64]. These processes will variably alter their size, morphology, crystallinity, surface charges, hydrophilicity and external contaminants. However, the present investigation shows that the original mineralogy of the silica and its host material may also significantly influence its toxicity. Characterisation of these parameters can therefore shed light on the variable toxicity of respirable crystalline silica.

## Conclusions

This study has shown that the surface properties and, therefore, toxicity of volcanic cristobalite particles are at least partly controlled by their geological origin. The results provide a compelling explanation for the anomalous toxicology data for cristobalite-rich volcanic ash and also highlight that inherent characteristics of industrial silica should be further studied. If RCS could be classified by its surface chemical composition and structure in relation to the factors that modify its hazard, then the single silica Occupational Exposure Limit (or Threshold Limit Value) currently adhered to across all industries could be modified to more closely represent the actual hazard of a particular RCS exposure.

## Methods

Theory 1 (crystal composition) was addressed by electron microprobe (Cameca SX100 wavelength dispersive electron probe microanalyser (with PeakSight software) at the University of Cambridge, UK. See Additional file 1 for detailed methods) to determine the composition of vapour-phase ‘prismatic’ (*n* = 55) and ‘platy’ (*n* = 8) cristobalite crystals, devitrification quartz (*n* = 4) and magmatic quartz crystals (for comparison) (*n* = 2). Only data with totals of 100 ± 0.8% were used in the study, as is common practice for anhydrous rock studies. Dome rock was used to provide large, flat, polished areas of crystals for analysis, which are harder to obtain from ash particles. The statistical difference in the compositions of prismatic and platy cristobalite crystals was determined using Welch’s two-sample *t*-test.

To address theories 2 (heterogeneous surface) and 3 (annealed surface), we adopted two strategies: 1) imaging minerals in thin sections of dome rock to determine the association of cristobalite with other local minerals; 2) imaging individual cristobalite ash particles in cross section to distinguish a cristobalite ‘core’ and a potentially nano-scale ‘rim’ of other minerals or glass.

Imaging of dome rock was carried out on carbon coated (30 nm) thin sections in BSE mode, and combined with elemental analysis on a LEO 1455VP SEM with Oxford Instruments INCA Energy Dispersive (EDS) X-ray analysis system at the Natural History Museum, London (working distance = 14-15 mm, accelerating voltage = 20 kV).

Several methods were developed for thin sectioning individual volcanic cristobalite crystals and two were implemented here. See Additional file 1 for full details.

1) Individual cristobalite crystals were thinned by focussed ion beam in a dual beam FIB-SEM (FEI Helios Nanolab at Durham University) to make them electron transparent [65]. The rim (protected from the FIB by a platinum strip deposited by gas injection) was then analysed by TEM-EDS (JEOL 2100F FEG-TEM with Oxford INCAx-sight Si(Li) EDS software at 200 kV) over several 50 nm transects from the rim into the crystal. However, this time-intensive technique was not suitable for mass analysis of cristobalite crystals so just two cristobalite crystals were analyzed.

2) Cristobalite crystals were separated from ash sample MBA12/7/03 using heavy liquids and mounted in a resin block. The polished blocks were carbon coated (~30 nm) and analysed in the Hitachi SU-70 FEG-SEM at Durham University. High-resolution SEM allowed examination of hundreds of sections through cristobalite crystals with ~5 nm resolution. The Oxford Instruments EDS system (INCAx-act LN2-free analytical Silicon Drift Detector) allowed verification of particle elemental compositions e.g. cristobalite (almost entirely Si and O, with minor Al and Na), plagioclase feldspar (Si, O, Al, Na and Ca) and glass (Si, O and minor Al, K, Na, Ca and Fe), as defined by Horwell et al. [41] as well as compositional variations along ‘rim’ to ‘core’ transects (over a distance of 5-10 μm).

## Abbreviations

RCS: Respirable crystalline silica; IARC: International Agency for Research on Cancer; SEM: Scanning electron microscopy; TEM: Transmission electron microscopy; FIB: Focussed ion beam; FEG: Field emission gun; EDS: Energy dispersive X-ray spectroscopy; BSE: Backscattered electron; PDC: Pyroclastic density current; SHV: Soufrière Hills volcano, Montserrat.

## Competing interests

There are no financial or non-financial competing interests.

## Authors’ contributions

CH acquired funding, designed the study, acquired, analysed and interpreted data and wrote the manuscript. BW helped design the study, acquired, analysed and interpreted data and assisted with manuscript preparation. KD assisted with manuscript preparation, contributing specific expertise in toxicological work on RCS. JLB acquired data and assisted with manuscript preparation. DD acquired data and assisted with manuscript preparation. LB developed sample preparation techniques and assisted with manuscript preparation. All authors read and approved the final manuscript and to the three anonymous reviewers.

## Authors’ information

CH is the founding Director of the International Volcanic Health Hazard Network (http://www.ivhhn.org) and the UK Natural Dust & Health Network (http://www.dur.ac.uk/claire.horwell/ukndhn).

## Supplementary Material

Additional file 1 **Additional methods.** 1) Electron microprobe detailed methods; 2) Additional methods for producing sections through ash particles; 3) Method for separation of cristobalite from ash using heavy liquids. Click here for file

## References

[B1] International Agency for Research on Cancer Silica, some silicates, coal dust and para-aramid fibrils 1997 International Agency for Research on Cancer, Lyon

[B2] Health and Safety Executive Respirable crystalline silica - phase 1. Variability in fibrogenic potency and exposure-response relationships for silicosis 2002 Health and Safety Executive, Sudbury

[B3] Haklader ACD Roberts AL Transformation of quartz to cristobalite J Am Ceram Soc 1961 44 35 41 10.1111/j.1151-2916.1961.tb15344.x

[B4] Ghiazza M Gazzano E Bonelli B Fenoglio I Polimeni M Ghigo D Garrone E Fubini B Formation of a vitreous phase at the surface of some commercial diatomaceous earth prevents the onset of oxidative stress effects Chem Res Toxicol 2008 22 136 145 19093746 10.1021/tx800270g

[B5] Hemenway DR Absher MP Trombley L Vacek PM Comparative clearance of quartz and cristobalite from the lung Am Ind Hyg Assoc J 1990 51 363 369 10.1080/15298669091369790 2166428

[B6] Meldrum M Howden P Crystalline silica: variability in fibrogenic potency Ann Occup Hyg 2002 46 27 30 10.1093/annhyg/46.suppl_1.27

[B7] Driscoll KE The toxicology of crystalline silica studies in vitro Appl Occup Environ Hyg 1995 10 1118 1125 10.1080/1047322X.1995.10389105

[B8] Fubini B Legrand AP Health effects of silica The surface properties of silicas 1998 John Wiley & Sons Ltd, England, Chichester 415 464

[B9] Small C Naumann T Holocene volcanism and the global distribution of human population Environ Hazards 2001 3 93 109

[B10] Baxter PJ Bonadonna C Dupree R Hards VL Kohn SC Murphy MD Nichols A Nicholson RA Norton G Searl A Cristobalite in volcanic ash of the Soufriere Hills Volcano, Montserrat, British West Indies Science 1999 283 1142 1145 10.1126/science.283.5405.1142 10024235

[B11] Dollberg DD Bolyard ML Smith DL Evaluation of physical health effects due to volcanic hazards: crystalline silica in Mount St. Helens volcanic ash Am J Public Health 1986 76 53 58 10.2105/AJPH.76.Suppl.53 3004241 PMC1651698

[B12] Horwell CJ Williamson BJ Llewellin EW Damby DE Le Blond JS Nature and formation of cristobalite at the Soufrière Hills volcano, Montserrat: implications for the petrology and stability of silicic volcanic domes Bull Volcanol 2013 75 696

[B13] Horwell CJ Braña LP Sparks RSJ Murphy MD Hards VL A geochemical investigation of fragmentation and physical fractionation in pyroclastic flows from the Soufriere Hills volcano, Montserrat J Volcanol Geotherm Res 2001 109 247 262 10.1016/S0377-0273(00)00319-X

[B14] Horwell CJ Grain size analysis of volcanic ash for the rapid assessment of respiratory health hazard J Environ Monitor 2007 9 1107 1115 10.1039/b710583p 17909645

[B15] Horwell CJ Hillman SE Cole PD Loughlin SC Llewellin EW Damby DE Christopher T Controls on variations in cristobalite abundance in ash generated by the Soufrière Hills volcano, Montserrat in the period 1997-2010 Geol Soc Lon Mem Accepted

[B16] Castranova V Pailes WH Dalal NS Miles PR Bowman L Vallyathan V Pack D Weber KC Hubbs A Schwegler-Berry D Enhanced pulmonary response to the inhalation of freshly fractured silica as compared with aged dust exposure Appl Occup Environ Hyg 1996 11 937 941 10.1080/1047322X.1996.10389993

[B17] Fubini B Bolis V Giamello E The surface chemistry of crushed quartz dust in relation to its pathogenicity Inorg Chim Acta 1987 138 193 197 10.1016/S0020-1693(00)81222-0

[B18] Fubini B Giamello E Pugliese L Volante M Mechanically induced defects in quartz and their impact on pathogenicity Solid State Ionics 1989 32/33 334 343

[B19] Fubini B Wallace WE Papirer E Modulation of silica pathogenicity by surface processes Absorption on silica surfaces 1999 France: Mulhouse 645 664

[B20] Vallyathan V Castranova V Pack D Leonard S Shumaker J Hubbs AF Shoemaker DA Ramsay DM Pretty JR McLaurin JL Freshly fractured quartz inhalation leads to enhanced lung injury and inflammation in rats. Potential role of free radicals Am J Respir Crit Care Med 1995 152 1003 1009 7663775 10.1164/ajrccm.152.3.7663775

[B21] Horwell CJ Fenoglio I Ragnarsdottir KV Sparks RSJ Fubini B Surface reactivity of volcanic ash from the eruption of Soufrière Hills volcano, Montserrat, with implications for health hazards Environ Res 2003 93 202 215 10.1016/S0013-9351(03)00044-6 12963405

[B22] Checkoway H Hughes JM Weill H Seixas NS Demers PA Crystalline silica exposure, radiological silicosis and lung cancer mortality in diatomaceous earth industry workers Thorax 1999 54 56 59 10.1136/thx.54.1.56 10343633 PMC1745344

[B23] Hughes JM Weill H Checkoway H Jones RN Henry MM Heyer NJ Seixas NS Demers PA Radiographic evidence of silicosis risk in the diatomaceous earth industry Am J Respir CritCare Med 1998 158 807 814 10.1164/ajrccm.158.3.9709103 9731009

[B24] Park R Rice F Stayner L Smith R Gilbert S Checkoway H Exposure to crystalline silica, silicosis, and lung disease other than cancer in diatomaceous earth industry workers: quantitative risk assessment Occup Environ Med 2002 59 36 43 10.1136/oem.59.1.36 11836467 PMC1740205

[B25] Rice FL Park R Stayner L Smith R Gilbert S Checkoway H Crystalline silica exposure and lung cancer mortality in diatomaceous earth industry workers: a quantitative risk assessment Occup Environ Med 2001 58 38 45 10.1136/oem.58.1.38 11119633 PMC1740036

[B26] Horwell CJ Baxter PJ The respiratory health hazards of volcanic ash: a review for volcanic risk mitigation Bull Volcanol 2006 69 1 24 10.1007/s00445-006-0052-y

[B27] Martin TR Wehner AP Butler J Evaluation of physical health effects due to volcanic hazards: the use of experimental systems to estimate the pulmonary toxicity of volcanic ash Am J Public Health 1986 76 59 65 10.2105/AJPH.76.Suppl.59 3080911 PMC1651697

[B28] Cullen RT Jones AD Miller BG Donaldson K Davis JMG Wilson M Tran CL Toxicity of volcanic ash from Montserrat. pp. 55 2002 Edinburgh: Institute of Occupational Medicine 55

[B29] Wilson MR Stone V Cullen RT Searl A Maynard RL Donaldson K In vitro toxicology of respirable Montserrat volcanic ash Occup Environ Med 2000 57 727 733 10.1136/oem.57.11.727 11024195 PMC1739881

[B30] Martin TR Chi EY Covert DS Hodson WA Kessler DE Moore WE Altman LC Butler J Comparative effects of inhaled volcanic ash and quartz in rats Am Rev Resp Dis 1983 128 144 152 6307099 10.1164/arrd.1983.128.1.144

[B31] Wehner AP Dagle GE Clark ML Buschbom RL Lung changes in rats following inhalation exposure to volcanic ash for two years Environ Res 1986 40 499 517 10.1016/S0013-9351(86)80125-6 3732218

[B32] Lee SH Richards RJ Montserrat volcanic ash induces lymph node granuloma and delayed lung inflammation Toxicology 2004 195 155 165 10.1016/j.tox.2003.09.013 14751671

[B33] Mannetje A Steenland K Attfield M Boffetta P Checkoway H DeKlerk N Koskela R-S Exposure-response analysis and risk assessment for silica and silicosis mortality in a pooled analysis of six cohorts Occup Environ Med 2002 59 723 728 10.1136/oem.59.11.723 12409529 PMC1740236

[B34] Hincks TK Aspinall WP Baxter PJ Searl A Sparks RSJ Woo G Long term exposure to respirable volcanic ash on Montserrat: a time series simulation Bull Volcanol 2006 68 266 284 10.1007/s00445-005-0006-9

[B35] Health and Safety Executive A regulatory impact assessment (RIA) on proposals to reduce the UK occupational exposure limit for respirable crystalline silica (RCS) 2007 London: Health & Safety Executive

[B36] Brown GM Donaldson K Brown DM Bronchoalveolar leukocyte response in experimental silicosis: Modulation by a soluble aluminium compound Toxicol Appl Pharmacol 1989 101 95 105 10.1016/0041-008X(89)90215-9 2552617

[B37] Fubini B Surface chemistry and quartz hazard Ann Occup Hyg 1998 42 521 530 9838865 10.1016/s0003-4878(98)00066-0

[B38] Begin R Masse S Rola-Pleszczynski M Martel M Desmarais Y Geoffroy M LeBouffant L Daniel H Martin J Aluminium lactate treatment alters the lung biological activity of quartz Exp Lung Res 1986 10 385 399 10.3109/01902148609058289 3013607

[B39] Duffin R Gilmour PS Schins RPF Clouter A Guy K Brown DM Macnee W Borm PJ Donaldson K Stone V Aluminium lactate treatment of DQ12 quartz inhibits its ability to cause inflammation, chemokine expression, and nuclear factor-κB activation Toxicol Appl Pharmacol 2001 176 10 17 10.1006/taap.2001.9268 11578144

[B40] Donaldson K Stone V Duffin R Clouter A Schins R Borm P The quartz hazard: effects of surface and matrix on inflammogenic activity J Environ Pathol Tox 2001 20 109 118 11570668

[B41] Horwell CJ Sparks RSJ Brewer TS Llewellin EW Williamson BJ The characterisation of respirable volcanic ash from the Soufriere Hills Volcano, Montserrat, with implications for health hazard Bull Volcanol 2003 65 346 362 10.1007/s00445-002-0266-6

[B42] Deer WA Howie RA Zussman J An introduction to the rock forming minerals 1996 2 New York: Longman Scientific and Technical

[B43] Finch GI Quarrell AG Roebuck JS The Beilby Layer Proc R Soc Lon Ser-A 1934 145 676 681 10.1098/rspa.1934.0129

[B44] Williamson BJ Di Muro A Horwell CJ Spieler O Llewellin EW Injection of vesicular magma into an andesitic dome at the effusive–explosive transition Earth Plan Sci Lett 2010 295 83 90 10.1016/j.epsl.2010.03.027

[B45] Šolc I The optical determination of a surface layer on polished quartz plates Czech J Phys 1966 16 525 528 10.1007/BF01689820

[B46] Talbot JH Kempis EB Finely ground quartz: evidence against a `Disturbed' layer Nature 1960 188 927 929

[B47] Quinot E Cavelier C Merceron MO Surface chemistry and cytotoxic properties of silica Biomedicine 1979 30 155 160 226193

[B48] Clouter A Brown D Hohr D Borm P Donaldson K Inflammatory effects of respirable quartz collected in workplaces versus standard DQ12 quartz: particle surface correlates Toxicol Sci 2001 63 90 98 10.1093/toxsci/63.1.90 11509748

[B49] Wallace WE Harrison JC Grayson RL Keane MJ Bolsaitis P Kennedy RD Wearden AQ Attfield MD Aluminosilicate surface contamination of respirable quartz particles from coal mine dusts and from clay work dusts Ann Occup Hyg 1994 38 439 445 10.1093/annhyg/38.inhaled_particles_VII.439

[B50] Stone V Jones R Rollo K Duffin R Donaldson K Brown DM Effect of coal mine dust and clay extracts on the biological activity of the quartz surface Toxicol Lett 2004 149 255 259 10.1016/j.toxlet.2003.12.036 15093271

[B51] Jacobsen M Rae S Walton WH Rogan JM Walton WH The relation between pneumoconiosis and dust-exposure in British Coal Mines Inhaled Particles III. Volume 2 1971 Pergamon Press 903 917 4359158

[B52] Walton WH Dodgson J Hadden GG Jacobsen M Walton WH, McGovern B The effect of quartz and other non-coal dusts in coalworkers pneumoconioisis 1) Epidemiological studies Inhaled Particles IV 1977 Oxford Pergamon Press 669 689 198367

[B53] Seaton A Dick JA Dodgson J Jacobsen M Quartz and pneumoconiosis in coalminers Lancet 1981 318 1272 1275 10.1016/S0140-6736(81)91503-8 6118680

[B54] Horwell CJ Fenoglio I Fubini B Iron-induced hydroxyl radical generation from basaltic volcanic ash Earth Plan Sci Lett 2007 261 662 669 10.1016/j.epsl.2007.07.032

[B55] Bruch J Rehn S Rehn B Borm PJ Fubini B Variation of biological responses to different respirable quartz flours determined by a vector model Int J Hyg Environ Health 2004 207 203 216 10.1078/1438-4639-00278 15330388

[B56] Donaldson K Borm P The quartz hazard: a variable entity Ann Occup Hyg 1998 42 287 294 9729916 10.1016/s0003-4878(98)00044-1

[B57] Bolis V Fubini B Marchese L Martra G Costa D Hydrophilic and hydrophobic sites on dehydrated crystalline and amorphous silicas J Chem Soc Faraday T 1991 87 497 505 10.1039/ft9918700497

[B58] Williamson BJ Pastiroff S Cressey G Piezoelectric properties of quartz and cristobalite airborne particulates as a cause of adverse health effects Atmos Environ 2001 35 3539 3542 10.1016/S1352-2310(01)00121-2

[B59] Daniel LN Mao Y Wang TCL Markey CJ Markey SP Shi XL Saffiotti U DNA strand breakage, thymine glycol production, and hydroxyl radical generation induced by different samples of crystalline silica in vitro Environ Res 1995 71 60 73 10.1006/enrs.1995.1068 8757240

[B60] Elias Z Poirot O Daniere MC Terzetti F Marande AM Dzwigaj S Pezerat H Fenoglio I Fubini B Cytotoxic and transforming effects of silica particles with different surface properties in Syrian hamster embryo (SHE) cells Toxicol in Vitro 2000 14 409 422 10.1016/S0887-2333(00)00039-4 10963957

[B61] Porter DW Barger M Robinson VA Leonard SS Landsittel D Castranova V Comparison of low doses of aged and freshly fractured silica on pulmonary inflammation and damage in the rat Toxicology 2002 175 63 71 10.1016/S0300-483X(02)00061-6 12049836

[B62] Vallyathan V Shi XL Dalal NS Irr W Castranova V Generation of free radicals from freshly fractured silica dust. Potential role in acute silica-induced lung injury Am Rev Resp Dis 1988 138 1213 1219 2849348 10.1164/ajrccm/138.5.1213

[B63] Fubini B Davis JMG, Jaurand MC Which surface functionalities are implied in dust toxicity? Cellular and molecular effects of mineral and synthetic dusts and fibres Volume H 85 1994 Berlin-Heidelberg: Springer-Verlag 347 358 NATO ASI Series

[B64] Fubini B Bolis V Cavenago A Volante M Physicochemical properties of crystalline silica dusts and their possible implication in various biological responses Scand J Work Environ Health 1995 21 9 14 8929680

[B65] Lee MR Brown DJ Smith CL Hodson ME Mackenzie M Hellmann R Characterization of mineral surfaces using FIB and TEM: A case study of naturally weathered alkali feldspars Am Mineral 2007 92 1383 1394 10.2138/am.2007.2453

