# Supplementary material for: The structure of volcanic cristobalite in relation to its toxicity; relevance for the variable crystalline silica hazard
Source: Part Fibre Toxicol. 2012 Nov 19;9:44. doi: 10.1186/1743-8977-9-44 (PMC3574026; doi:10.1186/1743-8977-9-44)
Supplement: Additional file 1 — Additional methods. 1) Electron microprobe detailed methods; 2) Additional methods for producing sections through ash particles; 3) Method for separation of cristobalite from ash using heavy liquids. [file 1743-8977-9-44-S1.docx]

**Supplemental Material**

**Inherent characteristics and external factors influencing the respiratory toxicity of volcanic crystalline silica; relevance for the variable silica hazard**

Claire J Horwell, Ben J Williamson, Ken Donaldson, Jennifer S Le Blond, David E Damby, Leon Bowen

**Additional Methodological Detail:**

1. Electron Microprobe

The following standards were used: (calibrated element and calculated ‘theoretical’ detection limits, in element ppm, shown in parentheses): jadeite (Na, 450), corundum (Al, 350), quartz (Si, 850), periclase (Mg, 62), K-feldspar (K, 95), diopside (Ca, 90), rutile (Ti, 80), pure manganese (Mn, 370) and fayalite (Fe, 300). AV = 15 kV, beam current = 4 nA and 60 nA (for major elements and minor/trace elements respectively), beam diameter = 5 µm. Fewer platy crystals were analysed than fish-scale crystals due to the difficulties of finding sufficiently wide crystals (~> 15 µm) for analysis (in thin section, platy crystals are acicular).

1. Additional methods for producing sections through ash particles
2. **FIB-SEM preparation**. Cristobalite crystals were hand-picked from ash sample MBA12/7/03 (see Horwell et al. In prep. for details). Individual crystals were placed in a dual beam FIB-SEM (FEI Helios Nanolab at Durham University). After verification that the crystal was crystalline silica (SiO_2_) by SEM-EDS, a focussed ion beam (FIB) was used to thin the crystal until it was electron transparent. An ~ 3 µm platinum strip was first deposited, using a gas injection system, on the outer edge of the crystal to avoid beam damage of this section of ‘rim’. Within the FIB-SEM, the thinned crystal was transferred to a TEM grid.
3. **Ultra-microtome.** Respirable (< 4 µm) ash particles (sample MRA5/6/99; see Horwell et al. 2003 for details and Table 1), separated from bulk ash using a British Rema Minisplit Classifier, were suspended in strong resin (Quetol) and electron transparent sections (< 70 nm) created using a vibrating Diatome ultra-microtome diamond knife with a 35 degree wedge angle at the University of Cambridge. Sections were mounted on holey carbon TEM grids with a light coating of evaporated carbon for support. Despite varying the resin composition and strength, the cristobalite crystals proved too hard and either shattered or were plucked from the resin. They also damaged the knife and ultimately this technique was abandoned.
4. Separation of cristobalite from ash using heavy liquids

The ash was first sieved to generate a sub-sample with a particle size of between 45 and 90 µm. Particles smaller than ~ 45 µm do not efficiently separate using heavy liquids. The subsample was washed with distilled water to remove any dust from particle surfaces. Magnetic particles were removed from the subsample using a plastic-wrapped hand magnet. The subsample was then stirred into sodium polytungstate (SPT) solution. The SPT had been pre-prepared with a density of 2.5 g cm^-^³, allowing the cristobalite particles to float (density 2.33 g cm^-^³) whilst other mineral phases sank (quartz = 2.65, plagioclase ~ 2.6 to 2.8 and hornblende ~ 3 to 3.6 g cm-³, Deer et al. 1996). Density separation was carried out over a period of a least 12 hours, or until there was a clear separation between floats and sinks. The floats on the surface of the SPT were then lifted off using a spatula, transferred to a small beaker and then sonicated several times in deionised water. The material was analysed by SEM-EDS and found to consist of ~ 60 % cristobalite, with other particles containing some vesicular glass which had reduced their average density. The crystals were then mounted in a 30 mm resin block (Araldite resin (AY103) and hardener (HY956), 5:1 ratio) which was polished using 1 µm diamond slurry.

**References**

Deer WA, Howie RA, Zussman J. 1996. An introduction to the rock forming minerals. 2nd ed. New York: Longman Scientific and Technical.

Horwell CJ, Cole PD, Loughlin SC, Damby DE, Hillman SE, Christopher T, et al. In prep. Cristobalite content of ash generated by 15 years of activity of the Soufrière Hills volcano, Montserrat. J Volcanol Geotherm Res.

Horwell CJ, Sparks RSJ, Brewer TS, Llewellin EW, Williamson BJ. 2003. The characterisation of respirable volcanic ash from the Soufriere Hills Volcano, Montserrat, with implications for health hazard. Bulletin of Volcanology 65: 346-362.
